# Water, water everywhere: environmental DNA can unlock population structure in elusive marine species

**DOI:** 10.1098/rsos.180537

**Published:** 2018-08-08

**Authors:** Kim M. Parsons, Meredith Everett, Marilyn Dahlheim, Linda Park

**Affiliations:** 1 Under Contract to NOAA, National Marine Fisheries Service, Marine Mammal Laboratory, Alaska Fisheries Science Center, 7600 Sand Point Way NE, Seattle, WA 98115, USA; 2 Marine Mammal Laboratory, Alaska Fisheries Science Center, 7600 Sand Point Way NE, Seattle, WA 98115, USA; 3 Under Contract to NOAA, National Marine Fisheries Service, 2725 Montlake Boulevard E, Seattle, WA 98112, USA; 4 Northwest Fisheries Science Center, 2725 Montlake Boulevard E, Seattle, WA 98112, USA

**Keywords:** porpoise, environmental DNA, population genetics, cetacean, stock, next-generation sequencing

## Abstract

Determining management units for natural populations is critical for effective conservation and management. However, collecting the requisite tissue samples for population genetic analyses remains the primary limiting factor for a number of marine species. The harbour porpoise (*Phocoena phocoena*), one of the smallest cetaceans in the Northern Hemisphere, is a primary example. These elusive, highly mobile small animals confound traditional approaches of collecting tissue samples for genetic analyses, yet their nearshore habitat makes them highly vulnerable to fisheries by-catch and the effects of habitat degradation. By exploiting the naturally shed cellular material in seawater and the power of next-generation sequencing, we develop a novel approach for generating population-specific mitochondrial sequence data from environmental DNA (eDNA) using surface seawater samples. Indications of significant genetic differentiation within a currently recognized management stock highlights the need for dedicated eDNA sampling throughout the population's range in southeast Alaska. This indirect sampling tactic for characterizing stock structure of small and endangered marine mammals has the potential to revolutionize population assessment for otherwise inaccessible marine taxa.

## 1. Introduction

As apex predators, marine mammals play a critical role as indicators of the health of the marine ecosystem [1,2]. This is particularly true for cetaceans (whales, dolphins and porpoises) that inhabit coastal waters and are susceptible to the direct impacts of numerous anthropogenic activities. Population genetics provides a means of generating key parameters for characterizing populations and facilitating conservation and management objectives by enabling the long-term monitoring of local populations. For species suffering mortalities from anthropogenic sources, determination of population structure (i.e. the size and range of the relevant management unit) is critical to understanding conservation status and developing appropriate mitigation strategies.

Accurate characterization and quantification of the genetic diversity within and among populations hinges upon the collection of an adequate number of samples representing the full genetic diversity across the geographical range of a population. While remote collection of tissue samples using biopsy darts has become a routine and highly successful method for many large cetacean species, efforts to collect significant numbers of genetic samples from some of the smaller, more enigmatic dolphin and porpoise species are still often restricted to beach-cast carcasses and fisheries by-catch. Such opportunistic samples provide insight into population structure and geographical breaks in genetic diversity, but lack of certainty about sample provenance and the infrequency of sample discovery present challenges for addressing questions pertaining to human-mediated environmental impacts on relatively local scales.

With the sole exception of the vaquita (*Phocoena sinus*), the harbour porpoise (*Phocoena phocoena*) is the smallest cetacean in the North Pacific. Harbour porpoise are broadly distributed through cold and temperate coastal waters along the continental shelf. The coastal habitat and prey preferences of this small, elusive marine mammal contribute to their vulnerability to anthropogenic impacts. In particular, their shallow coastal distribution makes them highly vulnerable to incidental capture during net-fishing operations [3–5]. The nature and magnitude of incidental takes are currently unknown but may be significant in some fisheries for Pacific salmon (*Oncorhynchus* spp.) and Pacific herring (*Clupea pallasi*) [6]. Line-transect abundance estimates from the inland waters of southeast Alaska indicated an overall decline in porpoise abundance in the mid-2000s. Contrasting trends between the northern and southern regions of this study area suggest significant population structuring within the currently recognized southeast Alaska stock [6]. Concern for localized impact on undefined harbour porpoise stocks underscores the need for population genetic analyses to better define population structure; however, sample sizes generated by both strandings and fisheries by-catch are severely limited in key geographical areas, and efforts to supplement these sources with remotely collected tissue biopsies have proved extraordinarily challenging for these small, elusive cetaceans. Because development of effective conservation and management strategies of living resources relies on our understanding of population dynamics and an accurate definition of genetic populations or stocks, generating the requisite genetic data despite obstacles to direct collection of tissue samples is a high priority [7].

To overcome these sampling challenges, we explored the use of seawater environmental DNA (eDNA) to obtain population-level genetic data. Exploiting the cellular material that is continually shed into aquatic environments by inhabiting organisms is increasingly being used to detect rare and invasive organisms [8,9] and to survey marine biodiversity [10–12]. Technological advances have led to rapid growth in this field and eDNA has been used in at least one case to generate population-level estimates of genetic diversity [13]. Using targeted seawater sampling, and taxon-specific genetic markers, we demonstrate the use of eDNA as a novel approach for characterizing the population genetic structure of harbour porpoise. Targeted amplicon sequencing and quantitative real-time polymerase chain reaction (PCR) provide insight into relative quantities of porpoise DNA in surface seawater samples, and generate mitochondrial sequence data that can be incorporated into a traditional framework for examining genetic diversity among harbour porpoise in the inland waters of southeast Alaska. This novel approach has the potential to unlock population structure in this and other elusive marine taxa where adequate sample sizes from direct sampling of individuals is limited.

## 2. Methods

### 2.1. Sample collection

Seawater samples for eDNA were collected in regions of historically high porpoise density [6] throughout the inland waters of southeast Alaska during two, two-week ship-based surveys in June and September 2016. When porpoise were sighted, an 8 m Zodiac was launched to enable close approaches and sample collection. Samples were collected from surface waters in the fluke prints within the wake of diving harbour porpoise using handheld, sterile 96 oz (approx. 3 l) wide-mouth collapsible Nalgene^©^ polyethylene canteens. Before the start of the field season, all collection canteens were sterilized with a 10% bleach solution and triple rinsed in distilled water to ensure they were free of any exogenous DNA. Samples were kept cool and dark until further processing in an enclosed bow box on the skiff. During sampling, the air and sea-surface temperatures (approx. 10°C) in Alaska were sufficiently low such that external cooling of the collected samples with ice packs was deemed unnecessary. Negative eDNA controls consisted of seawater samples collected in regions where no porpoise were observed.

Seawater eDNA samples were vacuum-filtered within 10 h of collection (mean = 3.42 h). The entire sample volume (2.3–3.1 l) was vacuum-filtered through a sterile, 0.45 µM nitrocellulose filter using a single-use filtration cup (Sterlitech, Auburn, WA) attached to a PVC vacuum filtration manifold. Post-filtration, filters were stored in 5 ml of Longmire's lysis buffer [14] at ambient temperature for storage and transport. The shipboard filtration area, as well as all equipment, including the filter manifold, forceps and gloves were treated with DNA AWAY™ (Molecular BioProducts, San Diego, CA) before the start of filtration to prevent contamination between samples or from other research operations taking place in the laboratory. All collection canteens were sterilized before and after every use, inside and out, by soaking for at least 10 min in a 10% bleach solution, followed by triple rinses with fresh tap water, and air drying.

### 2.2. DNA extraction

DNA extractions were carried out in a sterile biosafety cabinet in a laboratory dedicated to pre-PCR procedures. Rigorous controls for preventing and monitoring cross-sample contamination and contamination from exogenous DNA were employed throughout sample processing. Prior to each extraction set, the ventilation hood and all equipment were cleaned with DNA AWAY™ and irradiated with UV light for 30 min before and after each extraction. No harbour porpoise tissue extractions were performed concurrently during the period of eDNA extractions. Genomic DNA was extracted from each sample using a standard phenol–chloroform protocol [15]. Filters in Longmire's lysis buffer were incubated at 65°C for one hour on a shaking platform. eDNA from each sample was extracted in two paired extractions, one using 1 ml of Longmire's buffer, and the other using 1 ml of Longmire's buffer plus the eDNA filter. The extraction containing the filter was placed in a Qiagen Tissuelyser II (Qiagen, Germantown, MD) and shaken at an oscillation frequency of 25 Hz for 2 min 20 s with a single sterile, stainless steel 0.5 mm bead. After disruption, 1 ml of Longmire's lysis buffer was pipetted off and carried forward into the phenol–chloroform extractions, and the eDNA filter was frozen for archival purposes. Two chloroform extractions were performed as detailed in Sambrook & Russell [16] and Renshaw *et al*. [17]. After the second wash, 700 µl of the resulting aqueous layer was transferred to a clean 1.5 ml tube, 30 µl of 5 M NaCl and 700 µl of isopropanol were added to each sample and DNA was allowed to precipitate at −20°C overnight. After precipitation, samples were centrifuged for 20 min at 13 000 r.p.m. in a microcentrifuge to pellet DNA, ethanol removed and DNA resuspended in 100 µl of sterile, laboratory-grade water (Sigma-Aldrich, St Louis, MO). The two isolates from each sample were pooled and re-precipitated, and concentrated by adding 20 µl of 3 M sodium acetate and 400 µl of cold ethanol to each sample, inverting and precipitating overnight at −20°C to remove co-precipitated PCR inhibitors and residual salts. Samples were centrifuged for 20 min at 13 000 r.p.m. in a microcentrifuge, ethanol removed and DNA eluted in a final volume of 50 µl of sterile laboratory-grade water.

### 2.3. Harbour porpoise eDNA quantitative polymerase chain reaction

Quantitative PCR (qPCR) was used to detect and quantify harbour porpoise DNA in eDNA samples. Cytochrome *b* gene sequences for cetacean species known to co-occur with harbour porpoise in the inland waters of southeast Alaska were obtained from GenBank and aligned to identify species-specific regions for qPCR probe and primer design. A TaqMan^©^ probe specific to harbour porpoise (Ppho_Cytb-PROBE CTATGACTATTAGTAGTAAGAGCACCC) was designed for the target region and modified with 6-FAM reporter dye at the 5′ end and a non-fluorescent quencher at the 3′ end. Primers Ppho_Cytb-F (GTTCTTCATTTGTCTTTATATCCATATTG) and Ppho_Cytb-R (GCACCTCAAAATGATATTTGTCCT) were used to amplify a 160 bp region of the cytochrome oxidase *b* gene. Confirmation of species specificity was tested against the two most common odontocetes known to co-occur in the survey region, killer whale (*Orcinus orca*) and Dall's porpoise (*Phocoenoides dalli*), using DNA extracted from vouchered tissue samples (DNA provided by P. Morin, Southwest Fisheries Science Center, NOAA, National Marine Fisheries Service).

Real-time PCRs were performed on an Applied Biosystems 7900 in 10 µl reaction volumes containing 0.3 µM probe, 0.8 µM each primer, 1× TaqMan Environmental Master Mix 2.0 (Applied Biosystems) and 3 µl template eDNA. Following Applied Biosystems TaqMan protocol, thermal cycling conditions were set at 95°C for 10 min, followed by 50 cycles of 95°C for 15 s and 60°C for 1 min. Each set of reactions contained a minimum of two no-template controls to detect contamination with exogenous DNA. Relative standard curves (RSCs) were developed from 1 : 10 serial dilutions (1.3 ng µl^−1^ to 1 × 10^−5^ ng µl^−1^) of quantified target-species DNA extracted from a vouchered harbour porpoise tissue sample quantified on a Qubit™ (Invitrogen). The RSC slope and *y*-intercept were used to calculate the DNA concentration for the measured threshold cycle (*C*_t_) values.

### 2.4. Harbour porpoise eDNA primer development

Primer design for next-generation sequencing focused on the 5′ region of the mitochondrial control region. This section of the mitogenome is known to be hypervariable from ongoing genetic studies of North Pacific harbour porpoise [18,19] and captures phylogenetically informative sites. Primer design was based on a ClustalW alignment of 58 known North Pacific harbour porpoise haplotypes from sequence data generated from Sanger sequencing, in collaboration with the Southwest Fisheries Science Center, and from published sequences [20]. Primers were designed using NCBI's Primer-BLAST application [21] to capture the species-specific target region (approx. 450 bp). Each primer was tested *in silico* using the GenBank database (adjusting parameters for short input sequences), and *in vitro* with DNA extracted from vouchered tissues to confirm species specificity and ensure that the primers did not amplify the two commonly co-occurring, non-target odontocetes: killer whales and Dall's porpoise. PCR conditions were optimized to amplify the target fragment reliably in low copy number eDNA samples. PCR reactions were carried out in 10 µl volumes containing 2 µl of template eDNA, 1.2 mM MgCl_2_, 0.2 mM dNTP, 1.0 µM each forward and reverse primer (table 1), 2 µg µl^−1^ bovine serum albumin and 0.6 U Promega GoTaq (Promega, Madison, WI). PCR cycling conditions were as follows: 94°C for 2 min, followed by 40 cycles of 94°C for 1 min, 49°C for 1 min, 72°C for 1 min and a final extension of 5 min at 72°C.

**Table 1.** Harbour porpoise eDNA mitochondrial primers. Sequence in bold is the Illumina sequencing primer and adapter sequence.

primer name	sequence (5′-3′)
Ppho_Con1F	TAC TCC TTG AAA AAG CCC ATT GTA
Ppho_Con7R	ATG GTC CTG AAG TAA GAA CCA GAT G
Ppho_Con1F-Illumina	**TCG TCG GCA GCG TCA GAT GTG TAT AAG AGA CAG** TAC TCC TTG AAA AAG CCC ATT GTA
Ppho_Con7R-Illumina	**GTC TCG TGG GCT CGG AGA TGT GTA TAA GAG ACA G**AT GGT CCT GAA GTA AGA ACC AGA TG

### 2.5. Next-generation eDNA amplicon sequencing

Amplifications for Illumina sequencing were carried out in triplicate on all eDNA and positive control samples. Positive controls were generated using genomic DNA from four Sanger sequenced, vouchered harbour porpoise samples to test for PCR or sequencing error. DNA from each control sample was quantified using a Qubit™ 3.0 (ThermoFisher, Waltham, MA) and the Qubit DS Broad Range Assay (ThermoFisher, Waltham, MA) according to the manufacturer's instructions, and samples were normalized to a concentration of 5 ng µl^−1^. Replicate 10 µl reactions were performed for all eDNA and control samples, using optimized conditions (as above) and modified primers containing Illumina sequencing primer and adapter sequences. Each sample was amplified in three independent 10 µl reactions to ensure sufficient PCR product for next-generation sequencing. Products from all three PCRs were pooled and the presence of the desired amplicon size was confirmed by electrophoresis of the pooled 30 µl in a 2% agarose gel stained with SyberSafe (ThermoFisher, Waltham, MA). Amplicon size was determined with reference to a 100 bp DNA ladder (New England Biolabs, Ipswich, MA). Two negative control reactions were run in each set of PCR reactions, including template from one extraction blank and a no-template PCR sample. Bands from successful PCR of the eDNA samples and positive controls were gel excised using clean techniques to prevent cross-contamination between amplicons and purified using the Qiagen MinElute Gel Extraction spin-column protocol with a 30 µl elution volume (Qiagen, Germantown, MD). Gel electrophoresis of both negative controls confirmed the absence of detectable PCR product and these samples were not used for library generation.

Sequencing capture adapters with unique pairs of Illumina Nextera XT index tags were added to each sample using a second reduced-cycle PCR protocol according to the Illumina 16S metagenomic sequencing library preparation protocol as follows. This second PCR step was performed in 50 µl reaction volumes containing 8 µl of gel-purified PCR product from the first PCR, 5 µl each of one Illumina Nextera forward and reverse index tag, and 1× NEB Phusion High-Fidelity PCR Master Mix (New England Biolabs, Ipswich, MA). PCR cycling conditions were as follows: 95°C for 3 min, followed by 8 cycles of 95°C for 30 s, 55°C for 30 s, 72°C for 30 s, and a final extension of 5 min at 72°C. Indexed amplicons were normalized across samples using the SequalPrep Normalization Plate Kit (Applied Biosystems) and eluted in a 20 µl volume. Following normalization, indexed amplicons were pooled, cleaned using Ampure XP beads (Beckman Coulter, Brea, CA) and eluted in 25 µl of 10 mM Tris (pH 8.5). The indexed and pooled amplicon library was quantified via qPCR using the KAPA Library Quantification Kit for Illumina libraries (KAPA Biosystems, Wilmington, MA) according to the manufacturer's protocols and diluted to 4 nM. The final library was diluted to 12 pM and sequenced on an Illumina MiSeq Platform using the V3-600 paired-end sequencing chemistry (Illumina, San Diego, CA) with a PhiX spike-in of 10%.

### 2.6. Next-generation sequencing data analysis

The data analysis pipeline described below was first performed on sequence data for the four positive controls to calculate the expected rate of error generated during PCR and sequencing. Using data from each of the controls, we identified the most abundant sequence that had less than 100% identity to the known control region haplotype for that sample, and compared the read count to the total read count for the sample to derive an overall error rate due to non-specific priming and sequencing error [22]. The observed error rate was used to set a filter threshold for eDNA libraries; haplotypes appearing at a frequency below this threshold were removed from the data as described below.

Cutadapt (v. 1.13) [23] was used to trim Illumina adapters and primers from each sequence and quality filter each paired-end datafile separately using a threshold of -q 20. Read pairs were merged with VSEARCH (v. 2.4.3) [24] *fastq_mergepairs*, with a minimum overlap of 50 bp. VSEARCH was used to cluster identical sequences (or de-replicate), and restrict sequences to a minimum aligned length of 379 bp. Chimeric sequences were identified and discarded using the VSEARCH command *uchime denovo*, and sequences with a frequency of occurrence less than the empirically determined error rate (above) were removed from the analysis. Remaining sequences were compared to a local database of 58 known harbour porpoise control region haplotypes from the eastern North Pacific using the pairwise BLAST (Basic Local Alignment Search Tool) algorithm executed through the suite of command-line tools in the BLAST+ executables [25]. Sequences that failed to align to the local database were BLASTed against the NCBI database. Novel sequences were aligned in MAFFT [26] to confirm unique haplotypes. Phylogenetic relationships among all resolved haplotypes were explored using the program IQ-TREE [27]. The best model of nucleotide substitution and the maximum-likelihood tree were simultaneously inferred using the IQ-TREE algorithm using an alignment of all reference sequences trimmed to the 379 bp length of the eDNA consensus sequences. Branch support for maximum-likelihood trees was estimated using the ultrafast bootstrap approach implemented in IQ-TREE [28] using 5000 bootstrap replicates. Both Phi_ST_ and *χ*^2^ were used to generate preliminary estimates of genetic differentiation within southeast Alaska from mtDNA sequence data using the R package STRATAG [29].

## 3. Results

**Figure 1.**
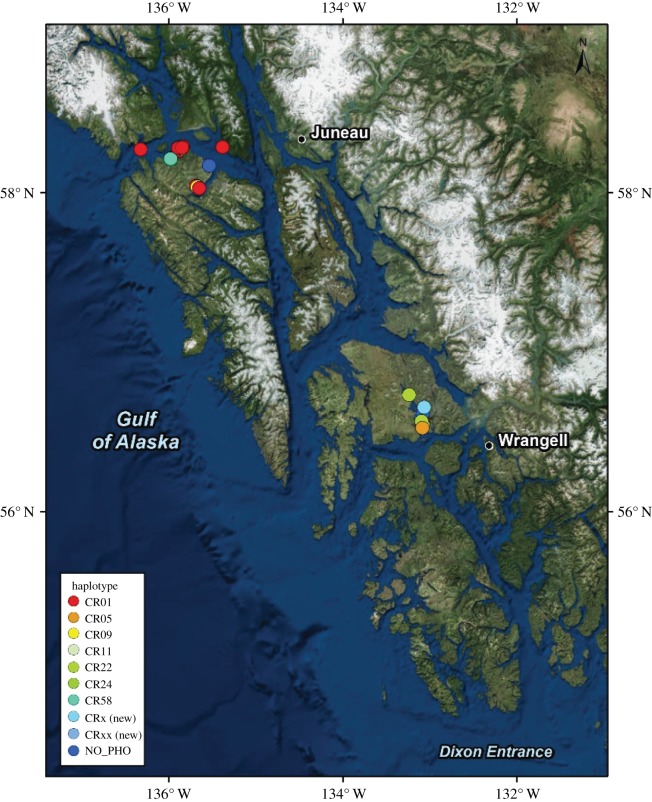
Environmental DNA (eDNA) sampling locations in southeast Alaska. Colours indicate mitochondrial control region haplotypes resolved from 36 eDNA samples (basemap source: Esri, DigitalGlobe, GeoEye, Earthstar Geographics, CNES/Airbus DS, USDA, USGS, AeroGRID, IGN, and the GIS User Community).

Forty surface seawater eDNA samples were collected during shipboard surveys in southeast Alaska inland waters on 10 different days between July and September 2016 (figure 1). The number of porpoise within visual range of the focal fluke print at the time of sample collection ranged from 1 to 15 (mean 3.95 ± 3.74). Harbour porpoise eDNA amplified in all 36 seawater samples collected from porpoise fluke prints. All field negative controls (*n* = 4) failed to amplify above the amplification threshold within 45 cycles with species-specific primers and the TaqMan probe. The median concentration of porpoise DNA for the 36 eDNA samples was 0.0033 ng µl^−1^, with values ranging from 8.96 × 10^−5^ to 0.112 ng µl^−1^ (average = 0.012 ng µl^−1^, s.d. = 0.022; electronic supplementary material). No cross-species amplification with harbour porpoise qPCR probes was detected in tests with genomic DNA from killer whale and Dall's porpoise.

A total of 34 874 482 raw paired-end reads were produced from a pooled library consisting of eDNA samples and positive controls, sequenced on a single Illumina MiSeq flow cell. The average number of reads passing filter per sample was 237 434.50 (s.d. = 74 914.37). Positive controls had an average of 101 643.75 reads after quality filtering, and between 93.78% and 95.56% of these reads represented the true, known control region haplotype for each sample. The maximum error rate for controls was 1.70% (average = 1.36%; s.d. = 0.29%) calculated based on the proportion of processed reads representing the most abundant sequence that was not identical to the known haplotype. This maximum error rate calculated from the control samples was carried forward in the analysis of eDNA samples; all de-replicated sequences representing less than 2% of processed reads were assumed to be either erroneous or non-target and eliminated from the dataset. All negative controls, including no-template PCR negatives, extraction blanks and four field negatives, failed to produce any amplification product detectable on the agarose gel.

Harbour porpoise control region sequences with a minimum length of 379 bp were successfully generated from 29 of 36 (80.56%) targeted eDNA samples. Gel-purified product from the libraries of seven samples failed to generate sequences of sufficient read depth to meet our stringent error rate criterion of greater than 2% of processed reads per sample, based on the analysis of sequences from tissue controls (above). The most abundant sequence for these eDNA samples either failed to align to any GenBank sequence, or aligned to the genome of a marine plankton (DQ257435) when BLASTed against the NCBI database. The total number of de-replicated reads for one sample (2016HP46) was atypically low (510) and although the sequence showed a high degree of similarity (99.74% identity) to a known harbour porpoise haplotype, it was not included as a newly discovered haplotype because the processed read depth relative to the total reads (221 922 merged pair contigs) was extremely low (less than 1% of all merged pair contigs) and may represent sequencing error.

**Figure 2.**
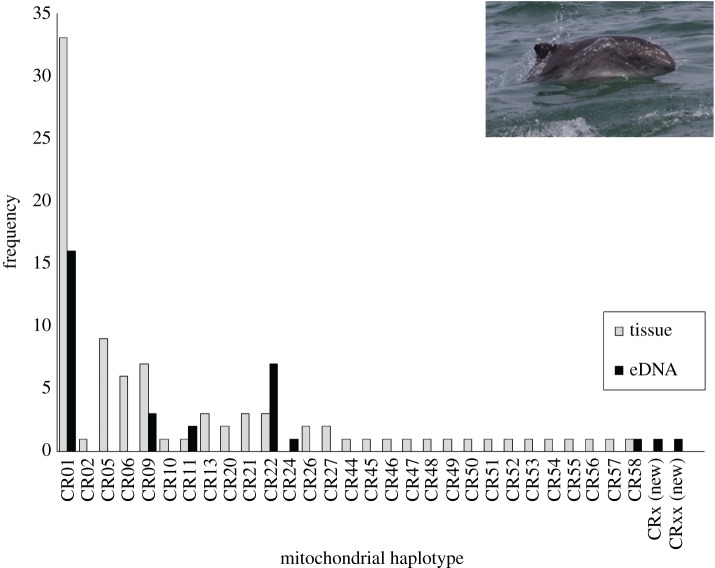
Frequency distribution of Alaska harbour porpoise control region (mitochondrial) haplotypes from tissue (*n* = 88; unpublished) and seawater eDNA (*n* = 36) samples (GenBank accession number available in electronic supplementary material, table S2). Photo credit D. Webster, NOAA National Marine Fisheries Service.

**Figure 3.**
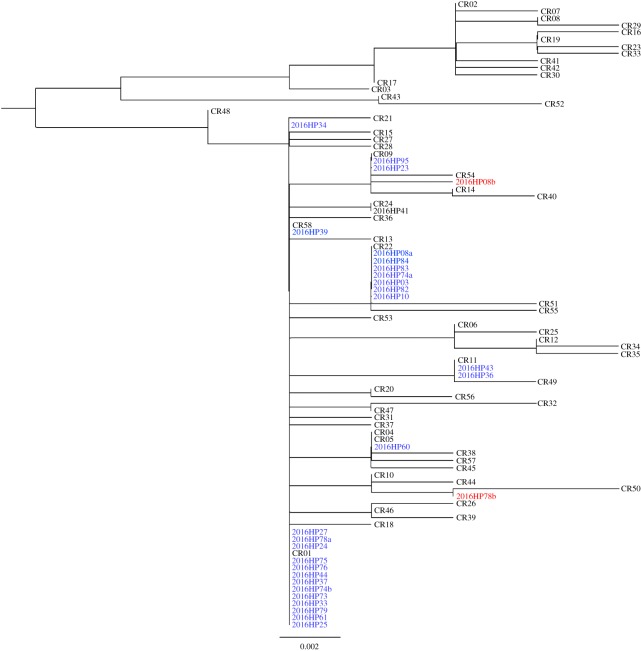
Unrooted maximum-likelihood bootstrap consensus tree based on the best fit substitution model (HKY_G4) determined by Bayesian information criteria. Known harbour porpoise haplotypes are indicated by CR01–CR58 (GenBank accession numbers available in electronic supplementary material, table S2). eDNA samples are denoted by blue text and samples with new haplotypes are labelled with red text. Sample names followed by ‘a' or ‘b' denote samples with multiple control region haplotypes.

Of the 29 eDNA samples that produced unequivocal harbour porpoise control region sequences, 26 samples yielded a single control region haplotype and three samples contained eDNA from multiple porpoise having two different haplotypes (electronic supplementary material). In all samples with multiple haplotypes, the less abundant haplotype was represented by greater than 20% post-processed reads. Eight unique haplotypes (based on a 379 bp fragment of the control region) were resolved from the eDNA samples, including two previously unreported haplotypes. Both were found in samples (2016HP08 and 2016HP78) that yielded multiple haplotypes (electronic supplementary material; figure 2). The high read depth (21 427 and 18 076, respectively) and the proportion of de-replicated sequences that comprised greater than 20% of post-processed reads (22.52% and 21.53%, respectively) suggest a very high probability that these secondary haplotypes are true control region haplotypes rather than sequencing error. The most common haplotype (CR01) occurred in 16 eDNA samples collected on 6 different days across the geographical survey range (figures 1 and 2). Of the eight mitochondrial haplotypes found in eDNA samples, five were previously known from tissue samples from Alaska harbour porpoise carcasses, and another had been previously identified from porpoise carcasses recovered in central and northern California (SWFSC 2017, unpublished). The most frequently observed haplotype in the eDNA samples was also the most common haplotype among a set of 88 tissue samples collected over multiple decades (1988–2016) from porpoise strandings and fisheries by-catch in Alaska waters (K. Parsons and P. Morin unpublished data, NOAA, National Marine Fisheries Service; figure 2). A phylogenetic tree based on the best fit substitution model (HKY_G4) and 28 informative sites across the 379 nucleotide sites highlights the similarity of the two new porpoise haplotypes from eDNA samples to previously known control region haplotypes (figure 3). Estimates of genetic differentiation (Phi_ST_ = 0.132, *p* = 0.022; *χ*^2^ = 16.24, *p* = 0.002) based on the geographical distribution of eDNA haplotypes suggest that genetic structure within southeast Alaska is likely.

## 4. Discussion

Identifying spatial genetic structure in natural populations is critical for defining management stocks and identifying region-specific susceptibility to anthropogenic and environmental stressors. Genetic data are particularly informative for marine vertebrates, such as cetaceans, that tend to be broadly distributed and capable of long-range dispersal. Characterizing this structure in harbour porpoise has, until now, been limited to the analysis of samples collected from beach-cast carcasses or fisheries by-catch; such sample types are typically restrictive because the rate of sample recovery from these opportunistic sources is low, and sample origin is often uncertain. The technological advances associated with next-generation sequencing have increased both the sensitivity and sequencing read depth, making it possible to generate mitochondrial sequence data from dilute target DNA. In the present study, we developed species-specific quantitative assays and generated mitochondrial haplotypes from eDNA seawater samples collected in the fluke prints of harbour porpoise. The high success rate of this approach and the amplification of sequence data that can contribute to reliable estimates of genetic diversity offer a valuable new avenue for generating key genetic data to guide the conservation and management of this small cetacean.

All surface seawater samples were collected in the fluke prints of diving porpoise in an effort to maximize the amount of target DNA in eDNA samples. Although the concentration of porpoise eDNA estimated through qPCR revealed considerable heterogeneity among samples (electronic supplementary material), harbour porpoise successfully amplified above the minimum amplification threshold from all eDNA samples. eDNA concentration has been correlated with the density and biomass of targeted species [22,30–32], and as such, eDNA sampling can be an informative addition to surveys aimed at estimating seasonality and distribution of aquatic species [9,22,33–36]. In addition to the biomass of target species, other biotic and abiotic factors are likely to affect the detectability of eDNA in seawater samples. A number of studies have attempted to examine the fate of eDNA in different aquatic environments and identify contributing factors that may act to preserve or degrade eDNA. Prolonged exposure to ultraviolet light [31], surface water temperature and pH [37,38], microbial community composition and the presence of tannins and other inhibitors of downstream PCR assays and the degradation of DNA in cellular material due to the time elapsed since deposition or shedding will all affect the quality and quantity of detectable eDNA in varied (and often unpredictable) ways [39,40]. Shedding rates of cellular material into the environment also appear to vary among species and among individuals in relation to behavioural states [37,38,41].

Designing assays to target organellar genomes, such as mitochondria, will help maximize the detection of target DNA in environmental samples due to the high copy number per cell compared to the nuclear genome [12]. Targeting mitochondrial gene regions maximizes the probability of detection not only due to the relative availability of target DNA per cell, but also extended persistence of detectable DNA in seawater due to the protection afforded by organellar membranes [42,43]. In the present study, we targeted regions of the mitochondrial genome for both qPCR and amplicon sequencing not only to maximize the probability of detection due to the inherent characteristics mentioned above, but also because the targeted 5′ section of the mitochondrial control region encompasses the specific sequence of interest used for past (and future) population genetic analyses. The ability to generate population-specific mitochondrial haplotypes from seawater samples dramatically expands future opportunities to examine the population genetic structure of small, threatened cetacean populations. Furthermore, the focused collection of seawater samples from porpoise fluke prints provides additional metadata useful to the specific needs of stock structure analyses because, unlike beach-cast strandings, the spatio-temporal attributes of each sample are known with greater certainty.

Harbour porpoise mtDNA haplotypes were generated from 80% of seawater samples and the difference in relative success rates between the real-time PCR and next-generation sequencing may reflect differences in the relative sensitivities of the two PCR-based approaches with greater sensitivity for the TaqMan™ probe in detecting low concentration target DNA [44]. Three of the 30 samples that generated harbour porpoise sequence data contained DNA from multiple porpoise with different haplotypes, with two different haplotypes resolved in each of the three samples. In all three multi-haplotype samples, one haplotype was dominant, comprising more than 59% of the read depth after quality filtering sequences (electronic supplementary material). In southeast Alaska, harbour porpoise group size varies seasonally, but typical groups range in size from singletons to groups of three individuals [45]. Seawater samples for the present study were collected in the fluke prints of a single porpoise, but the number of porpoises within the sampled group ranged from one to a maximum of 15. The persistence of eDNA in aquatic samples, and the spread of genetic material from its source organism is subject to a large number of variables; as such, it is difficult to predict with any certainty the number of species or organisms contributing genetic material to a given seawater sample [37,39,46]. Furthermore, while reliance upon mtDNA markers has many advantages, it also limits our ability to accurately estimate the number of genetic contributors within a given sample because of its unilateral mode of inheritance and highly conserved nature. Future development of methodologies that allow us to use species-specific nuclear DNA markers, such as single nucleotide polymorphisms, in combination with next-generation sequencing of eDNA will further enhance the value of these samples by allowing us to generate nuclear genotypes and apply models to estimate the minimum number of individuals within each seawater sample for target species.

We demonstrate here how high-throughput sequencing of seawater eDNA can generate mtDNA haplotypes for characterizing genetic diversity for marine vertebrate populations. Prior knowledge of haplotypic sequence diversity in Alaska harbour porpoise provided baseline data for comparison with eDNA haplotypes. Of the eight unique mtDNA sequences generated from eDNA samples, five were represented in the Alaska porpoise baseline dataset, with the most common eDNA haplotype also being the predominant haplotype from porpoise tissue samples (unpublished data). The two new haplotypes (CRx and CRxx) differed from two previously documented haplotypes (CR54 and CR50, respectively) by only a single nucleotide, and with representation by greater than 20% of final processed reads, these haplotypes were determined to be true genetic variants that have not yet been found in tissue samples from fisheries by-catch and stranded animals. While it is difficult to determine empirically whether or not the new haplotypes are true haplotypes or artefacts generated by early PCR error, resequencing of these samples and the collection of additional samples throughout the surveyed waters will help to determine the veracity of new sequence variants. The discovery of new haplotypes was not unexpected, considering the relatively high diversity of control region haplotypes and frequency of unique haplotypes found in single samples from the opportunistic tissue samples collected from Alaska harbour porpoise (figure 2). Further genetic sampling of harbour porpoise in inland Alaska waters will provide additional confirmation of these new haplotypes through discovery in additional tissue and eDNA samples.

Our goal was to examine whether population genetic data could be generated for small cetaceans through next-generation sequencing of eDNA samples. The high success rate and quality of mitochondrial sequences obtained by collecting surface seawater in the wake of focal animals offers a valuable alternative to traditional methods of collecting genetic samples that have proved to be largely ineffective for real-time biosampling of small, elusive cetaceans such as harbour porpoise. Amplicon sequencing using eDNA offers an approach that should allow the collection of a large number of samples and more thorough coverage of the surveyed geographical range, avoiding common complications in population genetic studies confronted by under-representation or bias in opportunistically collected samples. For example, Chivers *et al*. [18] noted that samples collected from stranded porpoise represented discrete locales within the population's range that did not necessarily correspond to the species' regional distribution, leaving some large numbers of porpoise essentially unsampled. In this case, disjunct sampled areas dictated *a priori* strata rather than true geographical breaks in the population's distribution. Although this limits inference of intraspecific population structure, significant genetic differentiation was detected between the two geographically sampled regions, warranting dedicated sampling throughout suitable habitat to identify significant population boundaries. In southeast Alaska, concentrations of harbour porpoise were consistently found in the same patchy regions throughout inland waters with concentrations in the northern and southern reaches [6]. If significant intraspecific structuring exists, as has been found in other regions [18,47,48], discontinuous distributions may indicate a highly stratified population. Smaller populations may not be able to withstand existing pressures of fisheries by-catch and other habitat altering anthropogenic activities, highlighting the need to determine biologically relevant management units.

Population genetics remains key for the conservation and management of natural populations, and contributes essential information needed to understand and mitigate human-mediated impacts on coastal marine species. This is particularly relevant for higher trophic, long-lived marine mammals which can serve as important barometers of the health of marine ecosystems [1,49–51]. The impacts of anthropogenic activities on cetacean populations can be complex and synergistic. While directed takes such as subsistence hunts can be measured empirically, other activities such as incidental takes and indirect effects due to environmental and prey impacts are best assessed by looking at temporal trends in population dynamics and changes in population size and structure. Genetic data provide a powerful tool for monitoring marine mammal populations, but collecting the requisite tissue samples from some of the smaller cetacean species can be problematic. Recent studies exploring the use of eDNA sampling for genetic monitoring of cetacean populations have demonstrated promise for future applications including detection and species identification [36,52–53]. This study represents a significant step in designing an approach to generate population genetic data for small and threatened marine vertebrates through eDNA methodologies.

## Supplementary Material

eDNA sample summary information.;Harbor porpoise control region haplotypes.

## Data accessibility

Unique control region haplotypes for Alaska harbour porpoise eDNA samples are available in Dryad at http://dx.doi.org/10.5061/dryad.77h8fd8 [54]. Upon acceptance, all mtDNA sequences will also be published in GenBank.
