# Supplementary material for: Water, water everywhere: environmental DNA can unlock population structure in elusive marine species
Source: R Soc Open Sci. 2018 Aug 8;5(8):180537. doi: 10.1098/rsos.180537 (PMC6124077; doi:10.1098/rsos.180537)
Supplement: eDNA sample summary information.;Harbor porpoise control region haplotypes. [file rsos180537supp1.docx]

Supplementary Data

Manuscript Title: Water, water everywhere: Environmental DNA can unlock population structure in elusive marine species.

Authors: Kim M. Parsons, Meredith Everett, Marilyn Dahlheim, Linda Park

Table I. Summarized results for all sequenced harbor porpoise tissue (control), seawater negative controls and seawater eDNA samples. ‘Percent reads represented by top sequence’ reflects the number of reads for the unique sequence with greatest frequency expressed as a percentage of the total number of reads passing quality control (QC) filters.


Table II. Genbank accession numbers for all eastern North Pacific harbor porpoise control region haplotypes referenced in this study.
